# Oral Single Dose Toxicity Study of Low Molecular Fucoidan in Mice

**DOI:** 10.5487/TR.2008.24.1.079

**Published:** 2008-03-01

**Authors:** Young-Mi Jung, Kang Min Yoo, Dong-Chan Park, Tae-Kwon Kim, Hyeung-Sik Lee, Sae-Kwang Ku

**Affiliations:** 111 ENZ Bio Co., Ltd., Daegu, 702-832 Korea; 211 Department of Genetic Engineering, College of Natural Sciences, Kyungpook National University, Daegu, 702-701 Korea; 311 Department of Herbal Biotechnology, Korea; 411 Department of Anatomy and Histology, College of Oriental Medicine, Daegu Haany University, 290, Yugok-dong, Gyeongsan-si, Gyeongsangbukdo, 712-715 Korea; 511 Development Team for The New Drug of Oriental Medicine (BK21 program), Daegu Haany University, Gyeongsan, 712-715 Korea

**Keywords:** Low molecular fucoidan, Single oral dose toxicity, Mice, Histopathology

## Abstract

This study was conducted to obtain information of the oral dose toxicity of low molecular fucoidan (LMF) in male and female mice. In order to calculate 50% lethal dose (LD_50_) and approximate lethal dose (LD), test material was once orally administered to male and female ICR mice at dose levels of 2000, 1000, 500, 250, 125 and 0 (vehicle control) mg/kg (body wt.). The mortality and the changes on body weight, clinical signs, gross observation and organ weight and histopathology of principle organs were monitored 14 days after LMF treatment. We could not find any mortalities, clinical signs, body weight changes and gross findings. In addition, significant changes in the organ weight and histopathology of principal organs were not observed except for some sporadic findings. The results obtained in this study suggest that LMF may not be toxic in mice and may be therefore safe for clinical use. The LD_50_ and approximate LD in mice after single oral dose of LMF were considered over 2000 mg/kg in both female and male mice.

## INTRODUCTION

Fucoidans, the sulfated polysaccharides extracted from brown algae, were first isolated almost one century ago, contain substantial percentages of L-fucose and sulfate ester groups (Berteau and Mulloy, 2003). Fucoidan has been extensively studied due to its numerous biological activities including anticoagulant and antithrombotic, antitumor, antiviral, anti-complement and anti-inflammatory activities (Patankar *et al.*, 1993; Béress *et al.*, 1993; Blondin *et al.*, 1996; Haroun-Bouhedja *et al.*, 2000; Bojakowski *et al.*, 2001; Marais and Joseleau, 2001).

Fucoidan is of particular pharmacological interest because its non-animal origin, exhibits anti-inflammatory activities and potent modulation of connective tissue proteolysis (Senni *et al.*, 2006). However, it is difficult to use because of its higher molecular weights; the absorption and bioavailability of high molecular weight fucoidan was relatively lower (Shimizu *et al.*, 2005). Therefore, it has been researched to reduce the molecular weights (Colliec *et al.*, 1991). Since the pharmacological effects of fucoidan were varied with molecular weights, fucoidan generally divided low (< 10 kDa), middle (10~10,000 kDa) and (> 10,000 kDa) high molecular fucoidan (Matsubara *et al.*, 2005). Low molecular fucoidan (LMF) also showed various pharmacological effects like high molecular weight fucoidan (HMF) (Zemani *et al.*, 2006; Alkhatib *et al.*, 2006; Lake *et al.*, 2006; Fréguin-Bouilland *et al.*, 2007).

Although preclinical studies using HMF have been performed (Li *et al.*, 2005); upto now, no detailed toxicological assessment of LMF has been reported. The objective of this study, therefore, was to obtain the primary safety information about LMF, obtained by acid hydrolysis of high molecular fucoidan extracts from brown seaweed according to a previously published protocol (Nardella *et al.*, 2000; Jung *et al.*, 2007).

## MATERIALS AND METHODS

***Experimental animals.*** Each of thirty female and male ICR mice (6-wk old upon receipt, SLC, Japan) was used after acclimatization for 11 days. The body weights of animals at receipt are female 25~27 g and male 28~30 g, respectively. Five animals were allocated per a polycarbonate cage in a temperature (20~25°C) and humidity (40~45%) controlled room. Light : dark cycle was 12 h : 12 h and feed (Samyang, Korea) and water were supplied free to access. All animals were overnight fasted (about 18 h) before dosing and terminal necropsy. Animals were marked by picric acid. The experimental protocols were conducted in accordance with internationally accepted principles for laboratory animal use and care as directed in the Korea Food and Drug Administration (KFDA) guidelines.

***Preparation of LMF.*** The LMF, prepared by ENZ Bio. Co. Ltd. (Korea), was obtained by acid hydrolysis of high molecular fucoidan extracts from brown seaweed according to a protocol previously patented (Nardella *et al.*, 2000; Jung *et al.*, 2007). Based on previously reported analytical methods (Dubois *et al.*, 1956; Farndale *et al.*, 1986), the characteristics of LMF were: weight-average molecular mass, 3 ± 0.5 kDa (polydispersity 1.9); fucose content 38.3% (w/w); galactose content 17.1% (w/w), uronic acid content 3% (w/w), sulfate content 28% (w/w), protein content 5.4% (w/w), moisture content 3.2% (w/w), and ash content 5% (w/w). Prepared LMF is light brownish-white powder, and stored in a desiccator to protect from light and humidity. The appearance of LMF in vehicle is light brownish-opaque but homogenous suspension at 200 mg/ml. It is well soluble below highest concentration; it showed clear light yellow solution in distilled water at 100, 50, 25 and 12.5 mg/ml concentration levels. The test article was single orally administered at a dosage volume of 10 ml/kg using distilled water as vehicle.

***Grouping and dosing.*** In KFDA (2005) and OECD (2001) guidelines, the recommended highest dose of test materials were 2000 mg/kg or the maximum solubility, and they also recommended that in case of acute toxicity in mice, the dosage volume were below 20 ml/kg. In the present study, the highest dose of LMF was selected as 2000 mg/kg because it was relatively well suspended or dissolved upto 200 mg/ml concentration in distilled water, and 1000, 500, 250 and 125 mg/kg was selected using common ratio 2. In addition, a vehicle control group was added. Animal was once orally dosed using a sonde attached to a syringe of 1ml after overnight fasting (about 18 h, water was not restricted). Food and water were further restricted for about 3 h after dosing. LMF was dosed at 10 ml/kg dosage levels in the present study.

***Observation of clinical signs.*** All abnormal clinical signs were recorded before and after dosing at least twice a day based on the functional observational battery test (Irwin, 1968; Dourish, 1987).

***Body weight changes.*** Body weights were measured at the day of dosing (Day 0) immediately before treatment, 1, 2, 7, 13 and 14 days after dosing.

***Necropsy.*** All animals were subjected to terminal necropsy. Animals were asphyxiated by carbon dioxide and gross necropsy was performed in all animals at Day 14 after overnight fasting (about 18 h, water was not restricted).

Specific organs grossly observed: lung, heart, kidney, spleen, testis, liver, pancreas, epididymis, popliteal lymph node, ovary, brain, and uterus.

***Organ weight measurement.*** The absolute organ weight was measured and then relative organ weight (% of body weight) was calculated for the following organs of all experimental animals when they were sacrificed.

Measured organs: lung, heart, kidney (left), spleen, testis (left), liver, pancreas (splenic lobes), epididymis (left), popliteal lymph node (left), ovary (left), brain, and uterus.

***Histopathology.*** Principle organs listed below were sampled at terminal necropsy, and fixed in 10% NBF (neutral buffered formalin). 18 h after fixation, paraffin embedding was conducted and 3~4 μm sections were prepared by routine histological methods. Representative sections of each specified organs were stained with Hematoxylin & Eosin for light microscopical examination.

Specific organs sampled: lung, heart, kidney (left), spleen, testis (left), liver, pancreas (splenic lobes), epididymis (left), popliteal lymph node (left), ovary (left), brain, and uterus.

***Statistical analyses.*** Changes of body weight were analyzed by Mann-Whitney U-Wilcoxon Rank Sum W test (MW test) compared to those of vehicle controls. $LD_{50}$ was calculated by Probit method. Statistical analy-

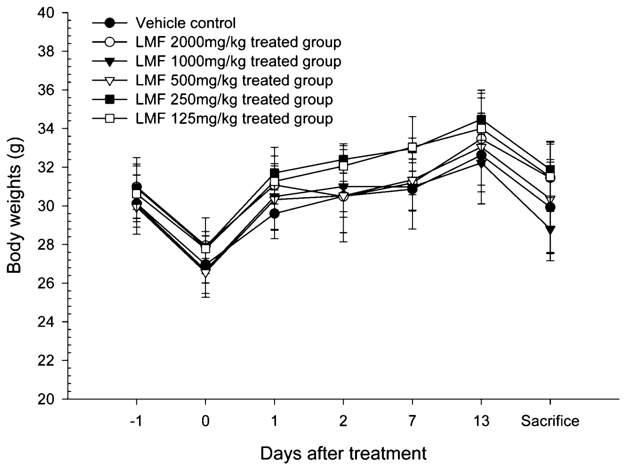

**Fig. 1.** Changes on the body weights in female mice during 14 days of observation after single oral treatment of LMF. No significant changes on the body weights were detected in LMF-treated groups as compared with vehicle control. F, overnight fasted.

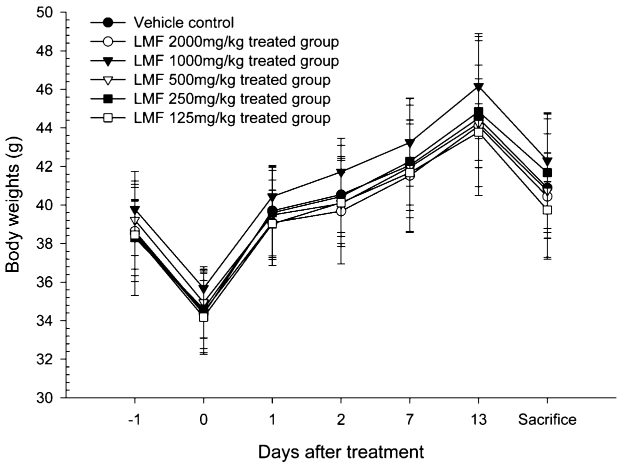

**Fig. 2.** Changes on the body weights in male mice during 14 days of observation after single oral treatment of LMF. No significant changes on the body weights were detected in LMF-treated groups as compared with vehicle control. F, overnight fasted.

**Table 1.** Changes in the absolute organ weights after oral dose of LMF

| Group | Organs: Male | | | | | | | | | | | |
|---|---|---|---|---|---|---|---|---|---|---|---|---|
| | Lung | Heart | Thymus | Kidney L | Adrenal gland L | Spleen | Testis L | Liver | Pancreas S | Brain | Epididymis L | Lymph node L[a] |
| Vehicle control | 0.212 ± 0.007 | 0.189 ± 0.009 | 0.061 ± 0.016 | 0.354 ± 0.044 | 0.010 ± 0.003 | 0.127 ± 0.010 | 0.120 ± 0.003 | 1.898 ± 0.177 | 0.222 ± 0.017 | 0.498 ± 0.014 | 0.048 ± 0.006 | 0.008 ± 0.002 |
| 2000 mg/kg | 0.209 ± 0.013 | 0.185 ± 0.008 | 0.067 ± 0.014 | 0.370 ± 0.032 | 0.009 ± 0.002 | 0.115 ± 0.019 | 0.122 ± 0.015 | 1.850 ± 0.091 | 0.220 ± 0.013 | 0.501 ± 0.015 | 0.049 ± 0.007 | 0.008 ± 0.002 |
| 1000 mg/kg | 0.214 ± 0.014 | 0.189 ± 0.015 | 0.067 ± 0.015 | 0.362 ± 0.042 | 0.009 ± 0.002 | 0.123 ± 0.016 | 0.125 ± 0.013 | 1.928 ± 0.185 | 0.243 ± 0.016 | 0.512 ± 0.029 | 0.050 ± 0.003 | 0.007 ± 0.002 |
| 500 mg/kg | 0.210 ± 0.015 | 0.202 ± 0.016 | 0.058 ± 0.015 | 0.353 ± 0.049 | 0.009 ± 0.001 | 0.104 ± 0.006 | 0.119 ± 0.007 | 1.833 ± 0.183 | 0.218 ± 0.022 | 0.499 ± 0.008 | 0.049 ± 0.005 | 0.010 ± 0.003 |
| 250 mg/kg | 0.207 ± 0.009 | 0.181 ± 0.016 | 0.073 ± 0.010 | 0.349 ± 0.059 | 0.010 ± 0.006 | 0.120 ± 0.021 | 0.113 ± 0.013 | 1.825 ± 0.172 | 0.242 ± 0.021 | 0.503 ± 0.010 | 0.044 ± 0.003 | 0.009 ± 0.003 |
| 125 mg/kg | 0.211 ± 0.009 | 0.183 ± 0.014 | 0.063 ± 0.014 | 0.349 ± 0.020 | 0.009 ± 0.002 | 0.112 ± 0.008* | 0.120 ± 0.022 | 1.746 ± 0.059 | 0.235 ± 0.021 | 0.511 ± 0.018 | 0.047 ± 0.007 | 0.008 ± 0.002 |
| | Organs: Female | | | | | | | | | | | |
| | Lung | Heart | Thymus | Kidney L | Adrenal gland L | Spleen | Ovary L | Liver | Pancreas S | Brain | Uterus | Lymph node L |
| Vehicle control | 0.182 ± 0.016 | 0.148 ± 0.013 | 0.063 ± 0.010 | 0.202 ± 0.016 | 0.009 ± 0.002 | 0.132 ± 0.038 | 0.033 ± 0.009 | 1.252 ± 0.178 | 0.178 ± 0.022 | 0.486 ± 0.005 | 0.152 ± 0.049 | 0.009 ± 0.004 |
| 2000 mg/kg | 0.182 ± 0.012 | 0.146 ± 0.011 | 0.066 ± 0.015 | 0.206 ± 0.011 | 0.009 ± 0.002 | 0.117 ± 0.013 | 0.033 ± 0.004 | 1.227 ± 0.070 | 0.178 ± 0.015 | 0.489 ± 0.015 | 0.201 ± 0.105 | 0.010 ± 0.003 |
| 1000 mg/kg | 0.184 ± 0.019 | 0.148 ± 0.020 | 0.050 ± 0.013 | 0.204 ± 0.019 | 0.008 ± 0.001 | 0.176 ± 0.139 | 0.035 ± 0.019 | 1.174 ± 0.144 | 0.171 ± 0.031 | 0.484 ± 0.022 | 0.145 ± 0.035 | 0.012 ± 0.004 |
| 500 mg/kg | 0.184 ± 0.021 | 0.152 ± 0.012 | 0.066 ± 0.006 | 0.208 ± 0.021 | 0.008 ± 0.001 | 0.133 ± 0.020 | 0.035 ± 0.006 | 1.268 ± 0.162 | 0.197 ± 0.028 | 0.501 ± 0.051 | 0.108 ± 0.035 | 0.009 ± 0.003 |
| 250 mg/kg | 0.184 ± 0.008 | 0.151 ± 0.010 | 0.070 ± 0.011 | 0.208 ± 0.025 | 0.008 ± 0.002 | 0.168 ± 0.058 | 0.041 ± 0.011 | 1.353 ± 0.163 | 0.185 ± 0.009 | 0.486 ± 0.009 | 0.167 ± 0.025 | 0.013 ± 0.005 |
| 125 mg/kg | 0.189 ± 0.005 | 0.158 ± 0.014 | 0.059 ± 0.012 | 0.217 ± 0.023 | 0.008 ± 0.001 | 0.121 ± 0.016 | 0.042 ± 0.006 | 1.274 ± 0.120 | 0.182 ± 0.022 | 0.517 ± 0.012** | 0.096 ± 0.026 | 0.009 ± 0.001 |

Values are expressed as mean ± S.D., g (n = 5); L, left sides; S, splenic lobes; [a]Popliteal lymph node; *$p < 0.05$ and **$p < 0.01$ compared to that of vehicle control by MW test.

ses were conducted using SPSS for Windows (Release 6.1.3., SPSS Inc., USA). In addition, degrees of clinical signs, gross and histopathological findings were subdivided into 3 degrees: 3+ Severe, 2+ moderate, 1+ slight.

## RESULTS

***Mortalities.*** No unscheduled or LMF-treatment-related mortalities were detected at all dose levels tested in this study. At the end of the treatment, all of animals were survived at all dose levels tested including vehicle control.

***Clinical signs.*** In this study, no LMF-treatment-related abnormal clinical signs were observed during observation periods regardless of gender.

***Changes in body weights.*** No significant changes on body weights were detected in all dosing groups tested compared to that of vehicle control in all dose levels tested (Fig. 1, 2).

***Changes in the organ weight.*** No meaningful changes on the absolute and relative organ weight of 11 principle organs were observed in all dosing groups tested compared to that of vehicle control except for significant ($p < 0.01$ or $p < 0.05$) decrease of absolute spleen weight in 125 mg/kg-dosing male group, of relative weight of spleen in 500mg/kg-dosing male group and increase of absolute brain weight in 125mg/kg-dosing female group, respectively (Table 1, 2).

***Necropsy findings.*** In this study, no meaningful changes on the gross findings of 12 principle organs were observed in all dosing groups tested compared to that of vehicle control except for some sporadic findings such as congestion spots of lung, atrophy of thymus, spleen atrophy, cyst formation of kidney, focal hemorrhage of thymus or adrenal gland with somewhat increases of incidences of liver atypical white foci in and liver yellowish discolorization in female and male LMF-

**Table 2.** Changes in the relative organ weights after oral dose of LMF

| Group | Organs: Male | | | | | | | | | | | |
|---|---|---|---|---|---|---|---|---|---|---|---|---|
| | Lung | Heart | Thymus | Kidney L | Adrenal gland L | Spleen | Testis L | Liver | Pancreas S | Brain | Epididymis L | Lymph node L[a] |
| Vehicle control | 0.521 ± 0.048 | 0.464 ± 0.029 | 0.150 ± 0.035 | 0.867 ± 0.101 | 0.023 ± 0.006 | 0.311 ± 0.026 | 0.294 ± 0.018 | 4.652 ± 0.374 | 0.543 ± 0.024 | 1.223 ± 0.093 | 0.117 ± 0.015 | 0.018 ± 0.004 |
| 2000 mg/kg | 0.520 ± 0.050 | 0.457 ± 0.016 | 0.165 ± 0.027 | 0.917 ± 0.066 | 0.022 ± 0.006 | 0.285 ± 0.041 | 0.300 ± 0.018 | 4.587 ± 0.267 | 0.545 ± 0.022 | 1.244 ± 0.080 | 0.122 ± 0.015 | 0.019 ± 0.004 |
| 1000 mg/kg | 0.507 ± 0.032 | 0.446 ± 0.030 | 0.159 ± 0.044 | 0.854 ± 0.075 | 0.022 ± 0.005 | 0.290 ± 0.031 | 0.295 ± 0.014 | 4.551 ± 0.211 | 0.575 ± 0.023 | 1.216 ± 0.127 | 0.119 ± 0.010 | 0.016 ± 0.003 |
| 500 mg/kg | 0.516 ± 0.028 | 0.495 ± 0.018 | 0.143 ± 0.041 | 0.866 ± 0.105 | 0.023 ± 0.003 | 0.256 ± 0.006* | 0.294 ± 0.028 | 4.493 ± 0.302 | 0.535 ± 0.045 | 1.225 ± 0.044 | 0.122 ± 0.013 | 0.024 ± 0.007 |
| 250 mg/kg | 0.498 ± 0.021 | 0.435 ± 0.022 | 0.175 ± 0.023 | 0.835 ± 0.112 | 0.024 ± 0.012 | 0.287 ± 0.041 | 0.273 ± 0.048 | 4.380 ± 0.253 | 0.583 ± 0.063 | 1.212 ± 0.079 | 0.107 ± 0.007 | 0.022 ± 0.007 |
| 125 mg/kg | 0.531 ± 0.015 | 0.461 ± 0.018 | 0.158 ± 0.037 | 0.878 ± 0.039 | 0.022 ± 0.005 | 0.283 ± 0.023 | 0.302 ± 0.056 | 4.397 ± 0.154 | 0.594 ± 0.066 | 1.285 ± 0.034 | 0.119 ± 0.018 | 0.021 ± 0.006 |
| | Organs: Female | | | | | | | | | | | |
| | Lung | Heart | Thymus | Kidney L | Adrenal gland L | Spleen | Ovary L | Liver | Pancreas S | Brain | Uterus | Lymph node L |
| Vehicle control | 0.608 ± 0.019 | 0.497 ± 0.073 | 0.212 ± 0.034 | 0.679 ± 0.070 | 0.031 ± 0.005 | 0.438 ± 0.102 | 0.110 ± 0.022 | 4.176 ± 0.403 | 0.593 ± 0.042 | 1.633 ± 0.132 | 0.517 ± 0.187 | 0.030 ± 0.017 |
| 2000 mg/kg | 0.580 ± 0.039 | 0.447 ± 0.055 | 0.203 ± 0.056 | 0.628 ± 0.048 | 0.026 ± 0.004 | 0.358 ± 0.050 | 0.102 ± 0.014 | 3.750 ± 0.368 | 0.545 ± 0.070 | 1.494 ± 0.126 | 0.597 ± 0.260 | 0.030 ± 0.010 |
| 1000 mg/kg | 0.641 ± 0.088 | 0.518 ± 0.098 | 0.173 ± 0.039 | 0.715 ± 0.110 | 0.027 ± 0.005 | 0.622 ± 0.505 | 0.119 ± 0.063 | 4.078 ± 0.491 | 0.597 ± 0.138 | 1.686 ± 0.150 | 0.518 ± 0.141 | 0.043 ± 0.015 |
| 500 mg/kg | 0.606 ± 0.041 | 0.504 ± 0.045 | 0.220 ± 0.028 | 0.685 ± 0.059 | 0.028 ± 0.006 | 0.438 ± 0.049 | 0.114 ± 0.019 | 4.168 ± 0.188 | 0.652 ± 0.100 | 1.654 ± 0.149 | 0.355 ± 0.098 | 0.028 ± 0.009 |
| 250 mg/kg | 0.579 ± 0.028 | 0.476 ± 0.040 | 0.220 ± 0.035 | 0.650 ± 0.053 | 0.025 ± 0.007 | 0.525 ± 0.163 | 0.129 ± 0.030 | 4.236 ± 0.373 | 0.582 ± 0.043 | 1.526 ± 0.077 | 0.525 ± 0.089 | 0.042 ± 0.015 |
| 125 mg/kg | 0.601 ± 0.031 | 0.502 ± 0.031 | 0.190 ± 0.042 | 0.688 ± 0.043 | 0.025 ± 0.004 | 0.385 ± 0.050 | 0.135 ± 0.021 | 4.038 ± 0.169 | 0.577 ± 0.047 | 1.645 ± 0.069 | 0.305 ± 0.092 | 0.029 ± 0.005 |

Values are expressed as mean ± S.D., % (n = 5); L, left sides; S, splenic lobes; [a]Popliteal lymph node; *$p < 0.01$ compared to that of vehicle control by MW test.

**Table 3.** Necropsy findings after oral dose of LMF

| Group | Male | | | | | | Female | | | | | |
|---|---|---|---|---|---|---|---|---|---|---|---|---|
| | Vehicle control | 2000 mg/kg | 1000 mg/kg | 500 mg/kg | 250 mg/kg | 125 mg/kg | Vehicle control | 2000 mg/kg | 1000 mg/kg | 500 mg/kg | 250 mg/kg | 125 mg/kg |
| Lung | | | | | | | | | | | | |
| Normal | 4/5 | 4/5 | 4/5 | 4/5 | 4/5 | 4/5 | 3/5 | 5/5 | 4/5 | 5/5 | 5/5 | 5/5 |
| Congestion | 1/5 | 1/5 | 1/5 | 1/5 | 1/5 | 1/5 | 2/5 | 0/5 | 1/5 | 0/5 | 0/5 | 0/5 |
| Heart | | | | | | | | | | | | |
| Normal | 5/5 | 5/5 | 5/5 | 5/5 | 5/5 | 5/5 | 5/5 | 5/5 | 5/5 | 5/5 | 5/5 | 5/5 |
| Thymus | | | | | | | | | | | | |
| Normal | 4/5 | 5/5 | 4/5 | 4/5 | 4/5 | 5/5 | 5/5 | 4/5 | 3/5 | 5/5 | 5/5 | 3/5 |
| Atrophy | 1/5 | 0/5 | 1/5 | 1/5 | 1/5 | 0/5 | 0/5 | 1/5 | 2/5 | 0/5 | 0/5 | 1/5 |
| Focal hemorrhage | 0/5 | 0/5 | 0/5 | 0/5 | 0/5 | 0/5 | 0/5 | 0/5 | 0/5 | 0/5 | 0/5 | 1/5 |
| Kidney | | | | | | | | | | | | |
| Normal | 5/5 | 5/5 | 4/5 | 5/5 | 4/5 | 5/5 | 5/5 | 5/5 | 4/5 | 5/5 | 4/5 | 5/5 |
| Discolorization | 0/5 | 0/5 | 1/5 | 0/5 | 0/5 | 0/5 | 0/5 | 0/5 | 1/5 | 0/5 | 0/5 | 0/5 |
| Hypertrophy | 0/5 | 0/5 | 0/5 | 0/5 | 1/5 | 0/5 | 0/5 | 0/5 | 0/5 | 0/5 | 0/5 | 0/5 |
| Cyst | 0/5 | 0/5 | 0/5 | 0/5 | 0/5 | 0/5 | 0/5 | 0/5 | 0/5 | 0/5 | 1/5 | 0/5 |
| Adrenal gland | | | | | | | | | | | | |
| Normal | 5/5 | 5/5 | 5/5 | 5/5 | 5/5 | 5/5 | 5/5 | 5/5 | 4/5 | 5/5 | 5/5 | 5/5 |
| Focal hemorrhage | 0/5 | 0/5 | 0/5 | 0/5 | 0/5 | 0/5 | 0/5 | 0/5 | 1/5 | 0/5 | 0/5 | 0/5 |
| Spleen | | | | | | | | | | | | |
| Normal | 4/5 | 4/5 | 5/5 | 4/5 | 5/5 | 5/5 | 4/5 | 5/5 | 4/5 | 5/5 | 3/5 | 5/5 |
| Atrophy | 1/5 | 1/5 | 0/5 | 1/5 | 0/5 | 0/5 | 1/5 | 0/5 | 0/5 | 0/5 | 1/5 | 0/5 |
| Hypertrophy | 0/5 | 0/5 | 0/5 | 0/5 | 0/5 | 0/5 | 0/5 | 0/5 | 1/5 | 0/5 | 1/5 | 0/5 |
| Testis/Ovary | | | | | | | | | | | | |
| Normal | 5/5 | 5/5 | 5/5 | 5/5 | 5/5 | 5/5 | 5/5 | 5/5 | 5/5 | 5/5 | 5/5 | 5/5 |
| Liver | | | | | | | | | | | | |
| Normal | 5/5 | 4/5 | 4/5 | 3/5 | 3/5 | 4/5 | 5/5 | 5/5 | 5/5 | 4/5 | 2/5 | 5/5 |
| Discolorization | 0/5 | 0/5 | 0/5 | 1/5 | 0/5 | 0/5 | 0/5 | 0/5 | 0/5 | 0/5 | 1/5 | 0/5 |
| Atypical Foci | 0/5 | 1/5 | 1/5 | 1/5 | 2/5 | 1/5 | 0/5 | 0/5 | 0/5 | 1/5 | 2/5 | 0/5 |
| Pancreas | | | | | | | | | | | | |
| Normal | 5/5 | 5/5 | 5/5 | 5/5 | 5/5 | 5/5 | 5/5 | 5/5 | 5/5 | 5/5 | 5/5 | 5/5 |
| Brain | | | | | | | | | | | | |
| Normal | 5/5 | 5/5 | 5/5 | 5/5 | 5/5 | 5/5 | 5/5 | 5/5 | 5/5 | 5/5 | 5/5 | 5/5 |
| Epididymis/Uterus | | | | | | | | | | | | |
| Normal | 5/5 | 5/5 | 5/5 | 5/5 | 5/5 | 5/5 | 5/5 | 5/5 | 5/5 | 5/5 | 5/5 | 5/5 |
| Lymph node[a] | | | | | | | | | | | | |
| Normal | 5/5 | 5/5 | 5/5 | 5/5 | 5/5 | 5/5 | 4/5 | 5/5 | 4/5 | 5/5 | 4/5 | 5/5 |
| Hypertrophy | 0/5 | 0/5 | 0/5 | 0/5 | 0/5 | 0/5 | 1/5 | 0/5 | 1/5 | 0/5 | 1/5 | 0/5 |
| Others | | | | | | | | | | | | |
| Normal | 5/5 | 5/5 | 5/5 | 5/5 | 5/5 | 5/5 | 5/5 | 5/5 | 4/5 | 5/5 | 4/5 | 5/5 |
| Orbital sinus[b] | 0/5 | 0/5 | 0/5 | 0/5 | 0/5 | 0/5 | 0/5 | 0/5 | 1/5 | 0/5 | 0/5 | 0/5 |
| Biting wound | 0/5 | 0/5 | 0/5 | 0/5 | 0/5 | 0/5 | 0/5 | 0/5 | 0/5 | 0/5 | 1/5 | 0/5 |

Observed animals/total observed animals (n = 5); [a]Bilateral popliteal lymph node; [b]Unilateral suppurative inflammation.

dosing groups as compared with those of each vehicle controls. In addition, hypertrophy of popliteal lymph node and spleen were detected in LMF 1000 and 250 mg/kg-dosing female groups (Table 3).

***Histopathological findings.*** No LMF treatment-related changes on the histopathological findings of 12 principle organs were observed in all dosing groups tested compared to that of vehicle control except for some accidental findings such as congestion of lung, focal infiltration of inflammatory cells with/without necrosis in liver, focal thymus or kidney hemorrhages, cystic formation in kidney, edematous changes on the uterus, and hyperplasia of lymphoid cells in the popliteal lymph node. In case of 250 mg/kg of LMF-dosing male groups, the incidence of focal infiltration of inflammatory cells with/without necrosis in liver was increased as compared with male vehicle control (Table 4).

## DISCUSSION

In the present study, we examined the acute toxicity of single oral dose with LMF in female and male mice as part of the safety test and tried to further clarify their

**Table 4.** Histopathological findings after oral administration of LMF

| Group | Male | | | | | | Female | | | | | |
|---|---|---|---|---|---|---|---|---|---|---|---|---|
| | Vehicle control | 2000 mg/kg | 1000 mg/kg | 500 mg/kg | 250 mg/kg | 125 mg/kg | Vehicle control | 2000 mg/kg | 1000 mg/kg | 500 mg/kg | 250 mg/kg | 125 mg/kg |
| Lung | | | | | | | | | | | | |
| Normal | 4/5 | 5/5 | 4/5 | 5/5 | 5/5 | 5/5 | 5/5 | 5/5 | 5/5 | 5/5 | 5/5 | 5/5 |
| Congestion | 1/5 | 0/5 | 1/5 | 0/5 | 0/5 | 0/5 | 0/5 | 0/5 | 0/5 | 0/5 | 0/5 | 0/5 |
| Heart | | | | | | | | | | | | |
| Normal | 5/5 | 5/5 | 5/5 | 5/5 | 5/5 | 5/5 | 5/5 | 5/5 | 5/5 | 5/5 | 5/5 | 5/5 |
| Thymus | | | | | | | | | | | | |
| Normal | 5/5 | 5/5 | 5/5 | 5/5 | 5/5 | 5/5 | 4/5 | 5/5 | 5/5 | 5/5 | 5/5 | 5/5 |
| Focal hemorrhage | 0/5 | 0/5 | 0/5 | 0/5 | 0/5 | 0/5 | 1/5 | 0/5 | 0/5 | 0/5 | 0/5 | 0/5 |
| Kidney | | | | | | | | | | | | |
| Normal | 5/5 | 5/5 | 5/5 | 5/5 | 4/5 | 5/5 | 5/5 | 5/5 | 4/5 | 5/5 | 4/5 | 5/5 |
| Focal hemorrhage | 0/5 | 0/5 | 0/5 | 0/5 | 1/5 | 0/5 | 0/5 | 0/5 | 0/5 | 0/5 | 0/5 | 0/5 |
| Cyst | 0/5 | 0/5 | 0/5 | 0/5 | 0/5 | 0/5 | 0/5 | 0/5 | 1/5 | 0/5 | 1/5 | 0/5 |
| Adrenal gland left | | | | | | | | | | | | |
| Normal | 5/5 | 5/5 | 5/5 | 5/5 | 5/5 | 5/5 | 5/5 | 5/5 | 5/5 | 5/5 | 5/5 | 5/5 |
| Spleen | | | | | | | | | | | | |
| Normal | 5/5 | 5/5 | 5/5 | 5/5 | 5/5 | 5/5 | 5/5 | 5/5 | 5/5 | 5/5 | 5/5 | 5/5 |
| Testis/Ovary left | | | | | | | | | | | | |
| Normal | 5/5 | 5/5 | 5/5 | 5/5 | 5/5 | 5/5 | 5/5 | 5/5 | 5/5 | 5/5 | 5/5 | 5/5 |
| Liver | | | | | | | | | | | | |
| Normal | 3/5 | 5/5 | 4/5 | 3/5 | 2/5 | 5/5 | 2/5 | 5/5 | 4/5 | 4/5 | 3/5 | 3/5 |
| IF-NE* | 2/5 | 0/5 | 1/5 | 2/5 | 3/5 | 0/5 | 3/5 | 0/5 | 1/5 | 1/5 | 2/5 | 2/5 |
| Pancreas splenic | | | | | | | | | | | | |
| Normal | 5/5 | 5/5 | 5/5 | 5/5 | 5/5 | 5/5 | 5/5 | 5/5 | 5/5 | 5/5 | 5/5 | 5/5 |
| Brain | | | | | | | | | | | | |
| Normal | 5/5 | 5/5 | 5/5 | 5/5 | 5/5 | 5/5 | 5/5 | 5/5 | 5/5 | 5/5 | 5/5 | 5/5 |
| Epididymis left/Uterus | | | | | | | | | | | | |
| Normal | 5/5 | 5/5 | 5/5 | 5/5 | 5/5 | 5/5 | 4/5 | 3/5 | 4/5 | 3/5 | 4/5 | 5/5 |
| Edematous changes | -- | -- | -- | -- | -- | -- | 1/5 | 2/5 | 1/5 | 2/5 | 1/5 | 0/5 |
| Lymph node[a] | | | | | | | | | | | | |
| Normal | 5/5 | 5/5 | 5/5 | 5/5 | 5/5 | 5/5 | 5/5 | 5/5 | 4/5 | 5/5 | 5/5 | 5/5 |
| Lymphoid hyperplasia | 0/5 | 0/5 | 0/5 | 0/5 | 0/5 | 0/5 | 0/5 | 0/5 | 1/5 | 0/5 | 0/5 | 0/5 |

Observed animals/total observed animals (n = 5); [a]Bilateral popliteal lymph node; *IF-NE: Focal inflammatory cell infiltration with necrosis.

safety for clinical use. Well corresponded with previous toxicity study of high molecular fucoidan (Li *et al.*, 2005), we could not find any mortalities, clinical signs, changes in the body weight and gross findings. In addition, no LMF-treatment related abnormal changes on the organ weight and histopathology of principle organs except for some accidental or dose-independent sporadic changes.

Significant ($p < 0.01$ or $p < 0.05$) decreases of absolute spleen weight detected in 125 mg/kg-dosing male group, of relative weight of spleen detected in 500 mg/kg-dosing male group and increase of absolute brain weight detected in 125 mg/kg-dosing female group were also considered as not LMF-treatment related abnormal changes. They did not show any dose-relationships and did not show any histopathological changes in these organs. In addition, these organ weights were well corresponded to the normal ranges of same aged mice as previously (Plata and Murphy, 1972; Yamaguchi *et al.*, 1983). Especially, the change on the brain weight was restricted to the absolute weight not relative weight, thus this change was considered as secondary change from the body weight changes.

Congestion spots of lung, atrophy of thymus, spleen atrophy, cyst formation of kidney and focal hemorrhage of thymus/adrenal gland detected in the present study as gross findings, and congestion of lung, focal infiltration of inflammatory cells with/without necrosis in liver, focal thymus or kidney hemorrhages, cystic formation in kidney, edematous changes on the uterus, and hyperplasia of lymphoid cells in the popliteal lymph node detected as histopathological findings were considered as accidental findings because they were restricted in some dosing groups and in some case, they were also observed in vehicle control. In addition, they did not showed dose-dependency and most of them were rarely observed in normal mice (Lee *et al.*, 2005, 2006, 2007). The edematous changes of uterus are general signs related to the estrus cycles (Banks, 1986).

The atypical white foci and yellowish discolorization of liver detected in gross findings were revealed as focal necrosis with infiltration of inflammatory cells. Although the incidence of focal infiltration of inflammatory cells with/without necrosis in liver was increased in 250mg/kg of LMF-dosing male group, it considered as accidental findings because it did not showed any dose-dependency and more frequently encountered in female vehicle control than those of LMF treated female groups. Hypertrophy of popliteal lymph node and spleen detected in LMF 1000 and 250 mg/kg-dosing female groups with/without hyperplasia of lymphoid cells in lymph node were considered as secondary inflammatory changes from the suppurative inflammation of orbital sinuses or biting wounds, detected at gross observation in same animals, respectively.

Although the Hodge and Sterner (1949) classify the $LD_{50}$ of non-toxic materials, as those of were 5000~15000 mg/kg as indicated by US Environmental Protection Agency (1998), recently notified guidelines by KFDA (2005) and OECD (2001) recommended that the highest oral dose of test materials was 2000 mg/kg. In the present study, the $LD_{50}$ and approximate LD in mice after single oral dose of LMF were considered over 2000 mg/kg, respectively in both male and female.

From these results, oral gavage of LMF caused no serious toxic effect to the male and female mice upto 2000 mg/kg - the highest dosage tested in this study and may be therefore safe for clinical use.
